# Replanting the Birthing Trees to support Aboriginal and Torres Strait Islander parents and babies: protocol for developmental evaluation of a comprehensive culturally responsive, trauma-aware, healing-informed, continuity of care(r) model

**DOI:** 10.3389/fpubh.2025.1721107

**Published:** 2026-01-28

**Authors:** Catherine Chamberlain, Jacqui Sundbery, Leonie Segal, Jacynta Krakouer, Marcia Langton, Jillian Donnelly, Jayne Kotz, Ellen McEvoy, Maddy Lyon, Neve Mucabel-Bue, Amalia Karahalios, Paul Gray, Emmanuel Gnanamanickam, Caroline Atkinson, Kimberley A. Jones, Helen Henderson, Helen Herrman, Maedah Aboutalebi Karkavandi, Alison Elliott, Gina Bundle, Roz Walker, Trish Ratajczak, Bridgette Kelly, Shawana Andrews, Doseena Fergie, Susan Walker, Elise Davis, Judy Atkinson, Helen McLachlan, Pamela McCalman, Della Forster, Deb Bowman, Tess Bright, Helen Skouteris, Skye Stewart, Storm Henry, Kristen Smith, Campbell Paul, Kootsy Canuto, Jane Fisher, Kate Reynolds, Phillipa Reppington, Naomi Priest, Sally Kendall, Tracy Reibel, Julie Andrews, Dave Carmody, Adrienne Lipscomb, Maddison Bell, Christine Parry, Vanessa Russ, Shakira R. Onwuka, Rhonda Marriott

**Affiliations:** 1 Onemda Aboriginal and Torres Strait Islander Health and Wellbeing, Melbourne School of Population and Global Health, University of Melbourne, Melbourne, VIC, Australia; 2 Ngangk Yira Institute for Change, Murdoch University, Murdoch, WA, Australia; 3 Health Economics and Social Policy Group, University of South Australia, Adelaide, SA, Australia; 4 Justice and Society, University of South Australia, Adelaide, SA, Australia; 5 Centre for Epidemiology and Biostatistics, Melbourne School of Population and Global Health, University of Melbourne, Melbourne, VIC, Australia; 6 Methods and Implementation Support for Clinical and Health (MISCH) Research Hub, Faculty of Medicine, Dentistry, and Health Sciences, University of Melbourne, Melbourne, VIC, Australia; 7 Jumbunna Institute for Indigenous Education and Research, University of Technology, Sydney, NSW, Australia; 8 Adelaide EpiCentre (Centre for Clinical Epidemiology), University of Adelaide, Adelaide, SA, Australia; 9 We Al-li Pty Ltd, Goolmangar, NSW, Australia; 10 Orygen, Parkville, VIC, Australia; 11 Centre for Youth Mental Health, University of Melbourne, Melbourne, VIC, Australia; 12 The Bouverie Centre, La Trobe University, Melbourne, VIC, Australia; 13 Royal Women’s Hospital, Melbourne, VIC, Australia; 14 Poche Centre for Indigenous Health, University of Melbourne, Melbourne, VIC, Australia; 15 Department of Obstetrics and Gynaecology, University of Melbourne, Melbourne, VIC, Australia; 16 Centre for Community Child Health, Murdoch Children’s Research Institute, Melbourne, VIC, Australia; 17 Judith Lumley Centre, School of Nursing and Midwifery, La Trobe University, Melbourne, VIC, Australia; 18 Waminda, South Coast Women’s Health and Wellbeing Aboriginal Corporation, Nowra, NSW, Australia; 19 Health and Social Care Unit, Monash University, Melbourne, VIC, Australia; 20 Royal Children’s Hospital, Melbourne, Mental Health, VIC, Australia; 21 Flinders Health and Medical Research Institute, Flinders University, Adelaide, SA, Australia; 22 Global and Women’s Health, Monash University, Melbourne, VIC, Australia; 23 Western Australia Country Health Service, Perth, WA, Australia; 24 Centre for Health Services Studies, University of Kent, Canterbury, United Kingdom; 25 Gabra Biik Wurruwila Wutja Indigenous Research Centre, La Trobe University, Melbourne, VIC, Australia; 26 Victorian Aboriginal Child Care Agency, Preston, VIC, Australia; 27 School of Allied Health, Curtain University, Bentley, WA, Australia; 28 Boodjari Yorgas Midwifery Group Practice, East Metropolitan Health Service, Mount Nasura, WA, Australia

**Keywords:** Aboriginal, birth, perinatal, equity, trauma, healing-informed

## Abstract

**Background:**

Aboriginal and Torres Strait Islander people experience intergenerational trauma as a legacy of the impacts of colonisation. Replanting the Birthing Trees (RBT) aims to transform compounding cycles of intergenerational trauma and harm to positively reinforcing cycles of intergenerational nurturing and recovery for Aboriginal and Torres Strait Islander parents and babies. This paper describes the protocol for developmental evaluation of the culturally responsive, trauma-aware, healing-informed, continuity of care(r) model to support Aboriginal and Torres Strait Islander parents during the first 2000 days (pregnancy, birth and the first 5 years after birth).

**Methods:**

The RBT project will be conducted in partnership with seven health services across Victoria (Royal Women’s Hospital and Mercy Hospital for Women) and Western Australia (WA) [Armadale Hospital, Western Australian Country Health Service (Northam, Narrogin, Moora and Merredin)], Australia. The RBT project consists of five workstreams: a resource repository including support framework; culturally validated sensitive enquiry tools; workforce development and training; continuity of care(r) toolkit; and strategies to support families to stay together from the start. The Consolidated Framework for Implementation Research (CFIR) informs implementation strategies. Acceptability, feasibility, costs and effectiveness will be evaluated using mixed methods analysis of qualitative and quantitative data, collected using key stakeholder interviews; parent and service provider discussion groups and interviews; cost audit; knowledge, attitude and practice surveys; pre- and post-implementation outcome data; interrupted time series analysis of routinely collected administrative linked data for primary and secondary outcomes; and co-design workshops. Competitive funding and human research ethics committee approval were assessed against Indigenous research excellence criteria with protocols to ensure the cultural and emotional safety of participants and communities.

**Discussion:**

Participatory action research approaches are used to foster reflective cycles on data within the research process. Findings will be shared in project newsletters, plain language summaries, presentations and publications.

## Introduction

In Australia, Aboriginal and Torres Strait Islander ways of knowing, being and doing have fostered the development of physical, social, and emotional wellbeing in Aboriginal and Torres Strait Islander communities for millennia prior to European colonisation (1–3). However, colonisation, violence and discrimination have led to and reinforce harmful compounding cycles of intergenerational and complex post-traumatic stress disorder (complex trauma) (4). Complex trauma, associated with earlier exposure to severe, repeated threats or abuse (5), is a root cause of health inequities (6, 7) and an international public health priority (6, 8). The World Health Organisation (WHO) (9) provides a framework for understanding compounding intergenerational effects of trauma impacting Aboriginal and Torres Strait Islander people (4). Structural and interpersonal violence has led to increased early life exposure to childhood adversity, which can impair early development (10, 11). Socioeconomic disadvantage can compound this early adversity and lead to health-harming behaviours (12), poor health (13) and economic (14) outcomes, including adverse pregnancy outcomes (15). Critically, trauma can impact parents’ capacity to nurture their child, leading to intergenerational trauma (16–18). Compounding cycles of intergenerational trauma are exemplified in high and persistently rising proportions of Aboriginal and Torres Strait Islander children in Out of Home Care (OOHC) (19, 20).

Pregnancy, birth, and the transition to becoming a parent is a critical time when both risk and protective factors for trauma-related distress and recovery converge with unique life-course opportunities (21–24). During pregnancy, there is an increased risk for ‘triggering’ of trauma-related distress. For mothers, physiological and social changes in pregnancy and early parenthood, along with poor birth and infant outcomes, and the threat of child protection service involvement, can heighten trauma survival responses. However, the birth of a baby also offers a unique opportunity for healing and recovery, with the joy that comes from bonding and attachment. At this time we can Heal the Past by Nurturing the Future (7) and support parents to transform a ‘vicious’ cycle of trauma to a ‘virtuous’ cycle of nurturing (25) and recovery (24). Pregnancy is also the first time since childhood that many adults have regular and frequent scheduled contacts with health services, and evidence shows access for Aboriginal and Torres Strait Islander women is lacking (26). This offers a unique and timely opportunity to identify and support parents experiencing complex trauma. To be successful, this requires a highly skilled and well-supported workforce and system structural competence (27) to address complex social and emotional issues and ensure ‘safety’ in perinatal care extends to include holistic cultural, social and emotional safety. In a national survey of primary maternity care providers, 98% reported that trauma was a significant issue impacting Aboriginal and Torres Strait Islander parents; yet nearly half (43%) were ‘not satisfied’ with the ability of their service to address this (28). More recent studies confirm that Aboriginal women frequently perceive available health services as culturally unsafe and health professionals are often inadequately trained and underprepared to work cross-culturally (26).

### Aims and objectives

RBT aims to improve perinatal support for Aboriginal and Torres Strait Islander parents to transform compounding cycles of intergenerational trauma and harm into positively reinforcing cycles of intergenerational nurturing and recovery. This highly innovative project will build the infrastructure to enable seven services to provide a model of culturally responsive, trauma-aware, healing-informed, continuity of care(r) for Aboriginal and Torres Strait Islander parents during the perinatal period.

This paper outlines the conceptual model and protocol planned by a majority Aboriginal-led team to implement and conduct developmental evaluation of a comprehensive co-designed model using participatory action research, implementation science and a mixed methods developmental evaluation approach to assess acceptability, feasibility, costs and effectiveness of the model, while ensuring cultural, social and emotional safety for participants and communities.

## Methods and analysis

### Patient and public (community) involvement

RBT builds on extensive prior research, involving rigorous community co-design in the Healing the Past by Nurturing the Future (HPNF) project around what is needed to provide culturally responsive, trauma-aware, healing-informed, continuity of perinatal care, which has been woven together with two additional programs:

1. *HPNF-* is a model of trauma-integrated perinatal care, co-designed with Aboriginal and Torres Strait Islander communities and key partner organisations (29–41). The program objectives included improving trauma awareness, support, safe recognition and assessment in perinatal care for Aboriginal and Torres Strait Islander parents. Intervention Mapping (IM) was used - a planning approach which uses theory and evidence as foundations for taking an ecological approach to assessing and intervening in health problems and fostering community participation (21). The co-design activities included participation of over 500 stakeholders from more than 50 institutions (7). The team has completed four action research cycles to co-design a needs assessment and cultural safety framework (34); and an innovative culturally grounded model of trauma-integrated perinatal care. The model is currently being piloted in one rural site in Victoria. This model includes resources to improve awareness of complex trauma among healthcare staff and Aboriginal and Torres Strait Islander parents; a support framework for healthcare staff and parents to access culturally responsive, trauma-integrated care; advocacy to Support Aboriginal and Torres Strait Islander Families to Stay Together from the Start (SAFeST Start). It also includes a guide to sensitive enquiry and training for healthcare staff, to ensure that the benefits of identification, using a newly validated Aboriginal and Torres Strait Islander Complex Trauma and Strengths Questionnaire (ACTSQ) for culturally-grounded assessment for individual planning for Aboriginal and Torres Strait Islander families outweigh possible harms.
2. *Continuity of Carer Models* are a health service and system approach shown to reduce preterm births and improve perinatal survival (42, 43). These approaches enable care to be received from a single or small group of practitioners which is considered essential for establishing trusting relationships between service providers and parents, a fundamental pre-requisite for providing culturally responsive, effective trauma-aware, healing-informed support for parents experiencing trauma. Aboriginal and Torres Strait Islander women currently have limited access to midwifery continuity of carer models (16, 44). Even so, there are now several programs demonstrating the acceptability and effectiveness of continuity of carer models for Aboriginal and Torres Strait Islander parents (43, 45). This includes the Baggarrook midwifery group practice implemented at the Royal Women’s Hospital and the Nangnak Baban Murrup midwifery group practice implemented at the Mercy Hospital for Women, both of which have demonstrated improvements in parent experiences (46) and perinatal and infant health outcomes (47).
3. *Baby Coming You Ready? (BCYR)* centres around a strengths-based and healing-focused holistic digitised assessment for Aboriginal and Torres Strait Islander mothers-to-be/mothers and fathers (48–50). BCYR has been shown to generate trust, engagement, honest disclosure, relevant care-plans and a deepened therapeutic friendship between mothers, fathers and their health-care provider (50). The BCYR program includes mandatory training for healthcare providers supporting program integrity. The digital assessment is initiated in a stand-alone appointment early in antenatal care. Sensitive touch-screen images, Aboriginal voice-overs and skip logic guide both the mother, father and health-care provider through a holistic social, emotional, cultural and spiritual wellbeing ‘narrative inquiry’. The parent selects images they relate to, capturing strengths, supports and concerns. Reflecting on and prioritising these, they then design their own solutions.

**Figure 1:**
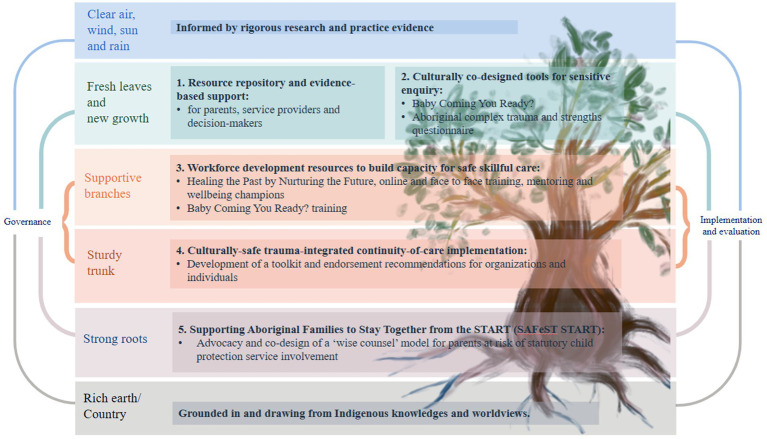
Replanting the Birthing Trees conceptual model.

RBT integrates elements of these distinct programs together into one comprehensive conceptual model for implementation across seven sites, as illustrated in Figure 1, and described in detail below.

The RBT governance structure reflects Aboriginal and Torres Strait Islander rights to self-determination as outlined in the United Nations Declaration on the Rights of Indigenous Peoples (Article 3) (51). In addition, it is aligned with the four priority reform pillars of the National Agreement on Closing the Gap (52), emphasizing: (1) formal partnerships and shared decision-making with governance group representation of Aboriginal peak bodies, chairing by the governance group by a senior Aboriginal and Torres Strait Islander Elder (DF), who receive four monthly reports from senior Aboriginal chief investigators (CC/RM); (2) building the community-controlled sector by embedding a policy-officer at the National Aboriginal Community Controlled Health Organisation (NACCHO) will ensure outcome delivery; (3) contributing evidence to transform government institutions, particularly health care provision and statutory child protection services; and (4) and prioritising access to and construction of data and information at a regional level, consistent with Indigenous Data Sovereignty Principles (53).

### Context and setting

RBT is being implemented and evaluated in two Australian jurisdictions (Victoria and WA) with seven health services (three metropolitan, four rural) from September 2023 to June 2026. The two participating sites in Victoria are the Royal Women’s Hospital (RWH; Baggarrook midwifery group practice) and the Mercy Hospital for Women (Nangnak Baban Murrup midwifery group practice). These are tertiary metropolitan services which proactively offer culturally responsive, continuity of midwifery care for >200 Aboriginal and Torres Strait Islander mothers per year.

The WA sites are Armadale Hospital in outer urban Perth, and four rural regional services managed by the WA Country Health Service Wheatbelt region including Avon Valley (Northam) and Narrogin Midwifery Group Practices, the Wheatbelt Aboriginal Health Service Aboriginal Liaison Officer – Maternal. Armadale Hospital offered Boodjari Yorga which provides Aboriginal-led midwifery and Aboriginal Health Worker (AHW) programs to approximately 130 women annually. Northam and Narrogin Health Services offer birthing care for low-risk births. Moora and Merredin Health Services offer antenatal/postnatal care, with transfer for birth to either Northam or Perth based secondary or tertiary maternity hospitals (impacts approximately 47% (35 of 75) of Wheatbelt Aboriginal women annually depending on assessed risk). These aforementioned WA services piloted the innovative BCYR perinatal program between 2021 and 2023. The successful mixed methods pilot included triangulated data analysis using women’s de-identified perinatal data (*n*=300) and interviews with women, midwives, nurses and managers.

These are ideal demonstration implementation sites for this project. Each includes (1) an exemplar model of culturally responsive continuity of midwifery care(r) for Aboriginal and Torres Strait Islander women/parents – an essential foundation for integrating trauma-aware, healing-informed care; (2) support for parents with complex social and emotional needs and trialling assessment tools; and (3) significant numbers of Aboriginal and Torres Strait Islander parents and infants seeking care. We have selected a mix of metropolitan and rural services to ensure the infrastructure and learning co-produced in this developmental evaluation project is more likely to be broadly applicable across a diverse range of settings, to build infrastructure for future scale-up and to evaluate implementation success and outcomes across settings.

### Methodological approach

RBT uses an overarching developmental evaluation (54) approach, consistent with action research methodology. We chose this model as it facilitates the adaptive development of change initiatives in complex and dynamic environments, and has been used in successfully in Aboriginal and Torres Strait Islander primary health care (54). It will enable the RBT teams to “respond to stakeholder feedback and apply learning in real-time to successfully refine theory-informed processes, tailor findings to stakeholders and context, and support the project’s dissemination and knowledge co-production aim - contributing to the production of robust, useable research findings for informing policy and system change” (54). Evaluation findings and modifications will be regularly discussed at monthly workstream and site implementation meetings, and four monthly investigator meetings and advisory group meetings. Decisions regarding adaptations to the predetermined conceptual model activities will be made by the investigator team with guidance and approval from the governance group; and adaptation to locally developed site implementation plans will be made by the site implementation teams. The governance group will receive reports of progress against all planned implementation activities, and intervene if there are concerns about the safety or progress with advice to the investigator team.

We will also use the RE-AIM evaluation approach to assess viability of future implementation at scale using multi-phase mixed methods. The RE-AIM Model describes the public health impact of an intervention as a function of five factors: reach, efficacy, adoption, implementation, and maintenance (55). This framework is consistent with systems-based and social-ecological thinking as well as community-based and public health interventions. A central premise of the RE-AIM Model is that the ultimate influence of an intervention is due to its combined effects on the above five evaluative factors.

### Implementation

Implementation will draw on the Diffusion of Innovations in Service Organisations conceptual model (56) and Consolidated Framework for Implementation Research (CFIR) (57). The distributive Diffusion of Innovations in Service Organisations conceptual model identifies the main factors that influence the uptake and implementation in organisations, to optimise diffusion, dissemination and sustainability (56). We will apply this model using the subsequently developed CFIR in our co-design approach ensuring that we achieve shared meanings and values, effective knowledge transfer, and to capture user-led innovation (56–58). An implementation and evaluation subgroup will use the CFIR to guide design of the RBT implementation methods. The CFIR has five major domains: intervention characteristics (e.g., evidence strength and quality), outer setting (e.g., patient needs and resources), inner setting (e.g., culture, leadership engagement), characteristics of the individuals involved, and the process of implementation (e.g., plan, evaluate, and reflect). The CFIR has been used successfully to bring together knowledge translation and participatory action research approaches in Aboriginal and Torres Strait islander healthcare (59).

### Conceptual model: integrating western and Indigenous worldviews and methodologies

The metaphor *Replanting the Birthing Trees* illustrates our innovative co-designed comprehensive model (Figure 1) aligned with the reform pillars of the National Agreement on Closing the Gap, grounded in Aboriginal and Torres Strait Islander knowledge and informed by research evidence to build the infrastructure for translation and scale-up. The model includes community-controlled governance and an implementation and evaluation working group using a developmental evaluation approach, with five core workstreams: -

1. A resource repository and support directory – development and delivery of resources for parents, service providers and decision-makers on culturally responsive, trauma-aware, healing-informed care. The minimum components will include sharing of parenting resources developed during this project as well as promotion of a ‘resource hub’ for parents and service providers in waiting areas, online and during training.
2. Optional implementation of culturally co-designed validated tools for sensitive enquiry: BCYR, ACTSQ. Partner organisations will consider the acceptability and feasibility of integrating these tools into their practice setting and whether to implement these.
3. Workforce development through creating resources to enable culturally responsive, trauma-aware, healing-informed care: and delivery of flexible online and face-to-face training, as well as mentoring for ‘wellbeing champions’ (HPNF and BCYR training). Each site will promote optional free online training to staff, and offer a number of one-day face to face training sessions dependant on the size of the organisation and staff availability. Each site will be offered one four-day face to face mentoring program. Delivery of all three training modalities will be considered implemented.
4. Culturally responsive, trauma-aware, healing-informed, continuity-of-care(r). A toolkit will be developed with partner organisations to support services which do not currently have continuity of care to implement these models. This will include a suite of standards of practise and tools, a core outcome set for evaluation, and exploration of options for organisational endorsement. Implementation will be considered as completion and distribution of the toolkit.
5. Supporting Aboriginal Families to Stay Together from the Start resources and advocacy. This includes co-designing a ‘wise counsel’ model of care for trauma-integrated, culturally responsive service provision and decision-making with Aboriginal and Torres Strait Islander organisations and community. This model aims to support Aboriginal and Torres Strait Islander parents and families interacting with child protection services during the perinatal period. Completion of a co-design workshop and accompanying manuscripts to provide guidance on developing a wise counsel model will be considered implementation.

The implementation of the workstream activities is designed to be flexible and responsive to the organisational context in line with the developmental evaluation approach. In addition to the predetermined workstream activities listed above, an implementation workshop will be held in each site to reflect on the findings of the pre-implementation findings from surveys and interviews. Services will have the opportunity to develop specific implementation plans to respond to identified needs. The anticipated timeline for implementation and evaluation activities is illustrated in Supplementary File 1.

We have developed a program logic that underpins the model and explains the proposed mechanism of impact on healthcare staff and families, as well as a logic model for evaluation, which corresponds to the research questions (Supplementary File 2). In sum, we expect that service providers involved in the project will increase their knowledge, confidence and practices in supporting Aboriginal and Torres Strait Islander families and that as a result of receiving culturally responsive, trauma-aware, healing-informed, continuity of care(r) perinatal models of care, we will see positive health and socio-emotional outcomes for parents and babies, including reductions in preterm births and reductions in the number of infants removed from families.

Site Implementation Teams (SITs) will be established to oversee implementation of the model as outlined in Figure 1 as well as additional local activities developed in the site implementation workshop, and foster local ownership and community participation, identify and incorporate relevant practice-based evidence and resources, address structural barriers, and oversee local adaptations. The SIT will maintain records of all implementation activities including barriers and enablers to implementation, service provider characteristics and participation in training (eligible, offered, completed). Consistent with the developmental evaluation approach, the implementation and evaluation activities will be adapted in each jurisdiction and site to respond to the context and stage each site is at with regards to the components of the overall model.

### Evaluation activities

The RBT project will assess the acceptability, feasibility, costs and effectiveness of a culturally responsive, trauma-aware, healing-informed, continuity of care(r) model for Aboriginal and Torres Strait Islander families. Methods to address each of the research questions are listed in Table 1. Consistent with a developmental evaluation approach, these activities will be adapted for each site based on feedback from key stakeholders. The primary and secondary outcomes for the overall study are detailed below under the description of secondary analysis of routinely collected linked administrative data using interrupted time series analysis.

**Table 1:** Research questions and overview of methods.

Research questions	Methods	Timing
1. What are the current practices in each of the seven sites to support Aboriginal and Torres Strait Islander women/parents during the antenatal/perinatal period?	Key stakeholder interviews (*n* = 21–56)	Pre-implementation and post implementation
2. What are the experiences of Aboriginal and Torres Strait Islander women/ parents receiving a culturally responsive, trauma-aware, healing-informed, continuity of care(r) model?	Yarning group with women/parents (*n* = 42–56)	Pre-implementation and 3-month post implementation
3. What is the organizational readiness to change and readiness for a trauma-informed approach? To what extent is the model of care implemented as intended? What modifications were made and why? What are the critical components of ‘good’ care? *(fidelity).* What are the barriers and enablers to implementation of a culturally responsive, trauma-aware, healing-informed, continuity of care(r) model (*feasibility*)?	Discussion groups/interviews with service providers (*N* = 56–70) *Service provider feedback portal	Pre-implementation (feasibility only); 3-month post implementation
4. What are the *costs and potential cost savings* of implementing a culturally responsive, trauma-aware, healing-informed, continuity of care(r) model? *(costs)*	Cost audit	During and post-implementation
5. How does the knowledge, attitudes and practices of service providers who support Aboriginal and Torres Strait Islander parents and babies change after training? *(effectiveness)*	KAP (Knowledge, attitudes and practices) survey with service providers (*N* = 350)	Baseline pre-training, post-training (during training), 3-month post-implementation
6. What is the impact on health outcomes for Aboriginal and Torres Strait Islander parents and babies? 6a Has this project reduced preterm births, NICU admissions, unborn notifications and OOHC admissions?	Secondary data analysis (*n* = 300/year in intervention sites plus control sites)	Post-implementation and post-evaluation
7. How can the data be interpreted and translated into action beyond this project?	Co-design workshops with service providers (*n* = 60 per workshop)	Implementation and post-implementation

Our approaches for recruitment and conducting culturally appropriate mixed methods research have been developed in collaboration with partners during extensive co-design. To maximise safety of participants, we will apply the cultural and emotional safety protocol developed during the co-design phase for this work (34). The research activities designed to address each of the research questions and to evaluate the implementation process and outcomes of the model are described here:

1. *Key stakeholder interviews.*

Research question: What are the current practices in each of the seven sites to support Aboriginal and Torres Strait Islander women/parents during the antenatal/perinatal period?

Recruitment and sample: We will conduct key stakeholder interviews for each site. Participants will be invited if they are aged 18 years or over, and employed in a role providing perinatal care to Aboriginal and Torres Strait Islander parents or in a management position related to maternity services, in the site area. Participants will be invited via flyers and information sessions conducted at sites and using snowballing techniques within the network. We estimate that approximately 20–50 interviews across the seven sites will be required to understand key contextual issues and reach saturation of themes. Data collection and analysis: Questions are aligned with the CFIR domains. Data from the interviews/discussion groups will be analysed using Braun and Clarke’s (2006) (35) six stages of thematic analysis to identify core themes, with reference to relevant theoretical frameworks. The audio recordings from the interviews/discussion groups will be transcribed and read by at least two researchers. Data involving Aboriginal and Torres Strait Islander people will be analysed by at least one Aboriginal and/or Torres Strait Islander researcher.

2. Yarning groups with parents

Research question: What are the experiences of Aboriginal and Torres Strait Islander women/ parents receiving a culturally responsive, trauma-aware, healing-informed, continuity of care(r) model?

Recruitment and sample: We will conduct pre-implementation and post-implementation yarning groups (60) with approximately 6–7 Aboriginal and Torres Strait Islander parents from each service (total 42–56) about their experiences of the perinatal care they received. Parents will be given information about the project via flyers and from service providers, and if interested, give permission to be contacted by the research team. Relational networks will also be utilised, with parents recruited through community and/or social networks as appropriate. We will give parents the option to participate either in a one-on-one interview or, if they prefer to bring a partner/family member/friend or support person along, we will offer a discussion group.

Pre-implementation, parents will be invited who are 16 years or older and identify as Aboriginal and/or Torres Strait Islander, can speak English and have a child five-years-old or younger and have received perinatal care at the hospital site previously. A post-implementation cohort of parents who gave birth since the start of implementation will be invited to participate three-months post implementation. There may be some parents who take part in both discussion groups.

Data collection and analysis: Discussions will be semi-structured with experienced interviewers sharing a story and using yarning methodology and broad open-ended questions. For example, “Thinking about the birth of your child: what were you hoping for?,” “What were the things that went well?,” and “If you were going back again, what would you change?” Parents will also be given the option of attending a creative expression session where they will have the opportunity to produce a creative piece of artwork, e.g., painting, drawing poem, which reflects their previous experience and responses to interview questions. For the post-implementation yarning group, additional questions will explore if they accessed any resources related to the project (i.e., BCYR program, resource repository), and their experiences with them (benefits or harms). Parents who participate will be given a supermarket voucher and small gift. Data will be analysed using thematic analysis outlined above with de-identified visual representations used to supplement the themes.

3. *Discussion groups/interviews with service providers and feedback portal*

Research question: What is the organizational readiness to change and readiness for a trauma-informed approach? To what extent is the model of care implemented as intended? What modifications were made and why? What are the critical components of ‘good’ care? (fidelity). What are the barriers and enablers to implementation of a culturally responsive, trauma-aware, healing-informed, continuity of care(r) model (feasibility)?

Recruitment and sample: Information sessions will be held at each service to provide an overview of the project and stages. The research team will explain the option to participate in the feedback and evaluation activities, including discussion groups. To achieve saturation of the themes we will conduct discussion groups/interviews with 6–10 service providers in each service (total ~56–70) aged 18 years or over who work in service site areas in a role associated with the delivery of perinatal care, express interest in participating and provide informed consent, before implementation and 3 months after implementation.

Data collection and analysis: Pre-implementation, the discussion group will include semi-structured interview questions (adapted version of the Barriers and Enablers to Trauma-Informed Care Implementation (BETICI)) (41) and a demographic questionnaire. Post-implementation, another 6–10 service providers will participate in a discussion group where they will be asked to describe the differences before and after implementation. Additional post-implementation questions will ask about what are the Most Significant Changes that have occurred, whether they have worked well (or not) and why. All staff at our seven implementation sites involved in perinatal care provision will also be invited to provide reflections on the project implementation via a feedback portal developed in REDCap. Staff who consent to take part in the feedback portal will be invited to fill out the feedback form related to positive or challenging examples. Service providers who complete face-to-face training will be invited by a follow up email to provide feedback via the portal. There will also be a link to the feedback portal on the site intranet and on flyers at sites. The feedback form will be anonymous to increase participation. Free text boxes will be provided for answers. All qualitative data will be analysed using thematic analysis outlined above. Quantitative data will be analysed using standard descriptive methods.

4. *Audit of service usage and cost post-implementation*

Research question: What are the costs and potential cost savings of implementing a culturally responsive, trauma-aware, healing-informed, continuity of care(r) model?

Data collection and analysis: The cost of implementation will draw on a thorough description of all program components, the specific activities involved, time allocated by the study/investigator team by occupation group (to which the appropriate hourly wage is applied), payment to contractors for services to support implementation, cost of consumables, including development of all materials, including parent information, training materials, service directory, delivery of materials, training of personnel – cost of trainer and time of attendees to determine a total program cost. Allocation of costs to families and children will be based on the number of families involved in the program delivery to allocate costs of consumables/recurrent costs, and for development costs (e.g., parent resources) using a 10-year timeframe, projected number of families who will ultimately access materials, and cost for resource updates.

To determine the additional costs of staff time spent completing training, mentoring, and reflective practice we will work closely with the SIT who will record this data.

Costing data will ultimately be combined with health outcome data in an economic evaluation that compares additional costs associated with the model of care to changes in health outcomes (cost-consequence analysis). The cost of scaling up and modelling forward will be based on a 10-year time frame and the regions to be covered drawing on program costings to inform the model parameters.

5. *KAP survey with service providers*

Research question: How does the knowledge, attitudes and practices of service providers who support Aboriginal and Torres Strait Islander parents and babies change after training?

Recruitment and sample: Service providers aged 18 years or over who work with parents in the sites and register for the HPNF face-to-face training program offered as part of Replanting the Birthing Trees will be asked to complete the KAP survey immediately before commencing training and again after the training session. Sample size calculations were informed by data from the HPNF pilot project, in which 500 participants completed the same training and KAP survey. In that project, mean KAP scores increased from 3.62 (SD = 1.08) at baseline (T1) to 3.80 (SD = 1.37) post-training (T2). Based on these parameters—and assuming an alpha of 0.05, statistical power of 0.90, a correlation of 0.5 between repeated measures, and a total population of 4,361 staff across both sites—a minimum sample size of 350 participants was calculated.

Data collection and analysis: The survey will include KAP items, demographic questions and a measure of job satisfaction. At follow-up, additional questions will be asked to assess satisfaction with the training, relevance of the training and improvements to the training. Given the hierarchical structure of the data, with providers nested within sites and repeated measures nested within providers, linear mixed-effects models (LMMs) will be employed to account for intra-individual correlations (within providers) and within-site correlations (between workplaces).

6. *Secondary analysis (using interrupted time series) of routinely collected linked administrative data*

Research question: What is the impact of this project on the following health outcomes for Aboriginal and Torres Strait Islander parents and babies: preterm births, NICU admissions, unborn notifications and OOHC admissions?

Recruitment and sample: We will conduct an interrupted time series (ITS) analysis to assess the effectiveness of the implementation of the model of care on health and socio-emotional outcomes. For the implementation site hospitals, we will use public hospital admitted patient records to identify all Aboriginal and Torres Strait Islander woman (and their babies) who have given birth at implementation site hospitals for two years before and after the implementation period and one year during the implementation period. We will also try to obtain a matched sample in a control group of hospitals that have not implemented the model of care to better understand if the post-implementation outcomes are due to the impact of the model or other factors.

Data collection and analysis: An interrupted time series (ITS) study of parent and infant outcomes. Secondary data will be linked across eight routinely collected health and social care administrative data sets, including: perinatal, emergency presentations, admitted patient, maternal and child health databases, birth registry, death registry, birthing outcomes, hospital separations and child protection databases. Data will be de-identified and linked by data custodians in Victoria and statisticians in WA based on the data generated by the external data linkage organisations. We will extract data related to all births among Aboriginal and Torres Strait Islander women (~300 per year across seven sites) for two years pre- and post-implementation and then aggregate the data to monthly intervals, so that we have 24 ‘points/months’ of data before the ‘interruption’ and 24 points/months’ of data after for ITS.

*Primary outcomes*: Proportion of children who are the subject of a protection (CP) notification(s) and proportion entering out-of-home care; antenatal visits (number, % of scheduled and % meeting all scheduled). Antenatal attendance is an indicator of how ‘safe’ parents’ feel.

Secondary outcomes: Routinely collected perinatal data: smoking; gestational weight gain greater or less than recommended; gestation; mode of birth; maternal and infant anemia; APGAR scores; birthweight; neonatal special care admissions; breastfeeding; child hospitalizations, still in hospital at 28 days. Due to the nature of the intervention, which is designed to strengthen organisational capacity and awareness, we expect a gradual change in slope rather than an immediate step change post-intervention. Segmented regression models will be fitted to estimate the change in trends before and after implementation (i.e., slope change).

7. *Co-design workshops*

Research question: How can the data be interpreted and translated into action beyond this project?

As we have done for the previous three national stakeholder workshops for previous phases of the Healing the Past by Nurturing the Future project held in Victoria, Northern Territory and South Australia, and BCYR stakeholder workshops in WA, we will invite approximately 60 key stakeholders to participate as co-researchers in three co-design workshops to enable broader collaboration in program development, implementation and evaluation.

Recruitment and sample: Key stakeholders are service providers, researchers, policymakers, and community leaders working to address complex trauma. Key stakeholders are identified through consultation and using snowballing during an ongoing process of advertising about the project through Aboriginal and academic health networks, professional meetings and conferences. People expressing interest in the project area continue to be included in an email list and receive updates about the project.

Data collection and analysis: The first workshop will involve refining the proposed ‘Wise Counsel’ model of care for parents and families involved with child protection services during the perinatal period. The second will refine implementation approaches, and the third workshop will discuss preliminary evaluation and outcome data and plans for dissemination. Data from the project will be presented in the workshops and workshop discussion data will be analysed descriptively (e.g., level of agreement with proposals) and using thematic analysis.

### Sample size calculations

The desired sample size for qualitative interviews/discussion groups has been selected noting the concept of saturation in qualitative research. Saturation is met when no new salient information is obtained from conducting further interviews. For quantitative data, all parents in the implementation and control sites giving birth two years before and two years after the intervention will be included in interrupted times series analyses, and data collected at baseline will enable estimation of the effect sizes in the ITS analysis. Sample size calculations for the pre- and post-training changes in knowledge, attitudes and practice are described previously.

### Confirming, renegotiating, discontinuation or withdrawal of participants from study

It will be clearly stated in all communications that the participant is free to withdraw from study at any time, for any reason, without prejudice, and with no obligation to give the reason for withdrawal. Participants will be informed that if they choose to withdraw from the study, the valid data provided will be deleted if it has not been deidentified at that point.

## Discussion

This comprehensive mixed methods implementation study aims to improve culturally responsive, trauma-aware and healing informed care for Aboriginal and Torres Strait Islander parents in the First 2000 days. We use a pragmatic participatory action research approach that utilizes existing data sources and collects implementation data that can be rapidly fed back to partner services to improve implementation.

This research builds on work currently being implemented with the HPNF project (63), and BCYR, Baggarrook midwifery group practice and the Nangnak Baban Murrup midwifery group practice programs (46, 47).

This is a rigorous mixed methods evaluation plan for a comprehensive intervention to improve outcomes for Aboriginal and Torres Strait islander families. However, there are limitations. We could not randomly allocate individuals to an intervention due to the organisation wide approach. Therefore there is the potential that changes in the primary and secondary outcomes may be due to effects other than the intervention. Furthermore, there may be variability in organisational readiness, workforce capability and leadership engagement which may influence fidelity.

We are mitigating this risk of bias by careful documentation and journalling of activities and potential confounders during the project on a timeline for consideration in the interrupted time series analyses, and utilising a control group for the secondary data analysis to evaluate if there were similar changes over time between intervention and control organisations. Applied research projects with rigorous evaluation of outcomes are urgently needed for Closing the Gap in health inequities which are disproportionately experienced by Aboriginal and Torres Strait Islander families. We anticipate that there will be many learnings from this implementation project about what works, what does not, and the barriers and enablers to improving care for Aboriginal and Torres Strait Islander families in both urban and rural settings.

## Ethics and dissemination

The funding proposal and human research ethics submission for this research was assessed against Indigenous research criteria developed to promote ethical and culturally appropriate research. This includes the National Health and Medical Research Council Ethical Guidelines for Research with Aboriginal and Torres Strait Islander people (61) and Australian Institute of Aboriginal and Torres Strait Islander Studies Code of Ethics for Aboriginal and Torres Strait Islander Research 2020 (62) which are grounded in the core rights to self-determination. We have received ethics approval for Victoria from the St Vincents Human Research Ethics committee (St Vincents HREC 148/23), Mercy Health HREC (Mercy HREC Ref 2023–032) and Western Australian Aboriginal Health Ethics Committee (HREC1297). We have developed an emotional and cultural safety protocol based on community consultation which will guide all project activities (34).

This implementation project is being conducted using participatory action research and implementation principles. Action research participative processes are embedded in the research design (e.g., workshops, and discussion groups). Other dissemination processes will include:

- Opportunity to comment on draft findings and provision of final findings to all participants in an academic and plain language format.
- Newsletter updates with project highlights and links to further information sent to all stakeholders three times a year.
- All project reports, summaries, presentations and newsletters provided on a designated project website.
- Presentations/in-service offered to partner organisation staff at community meetings.
- Presentations in conferences and Aboriginal and Torres Strait Islander research forums.
- All publications in open-access format with links available on the project website.
- Use of art, presentations and other culturally relevant mediums to share information.
- Policy briefs of relevant findings for policy developed with governance group.

Findings will be shared with the project implementation sites as available with a view to supporting the facilitation of continuous quality improvement processes. Parents and service providers who participate in any of the research activities will be given the opportunity when providing consent to indicate that they wish to receive a copy of the study findings in a community feedback format by providing either their email or postal address. At the conclusion of the study, those who have indicated they would like to be informed of the findings will be emailed/posted a plain language summary of the study findings.

### Strengths and limitations of this study

- This highly innovative project will build the infrastructure to enable seven services to provide a model of culturally responsive, trauma-aware, healing-informed, continuity of care(r) for Aboriginal and Torres Strait Islander parents during the perinatal period.
- Testing a comprehensive multi-component approach to generate reinforcing cycles of intergenerational nurturing and recovery for Aboriginal and Torres Strait Islander parents and babies.
- Best practice implementation science using consolidated frameworks and participatory approaches, with Aboriginal and Torres Strait Islander community-controlled governance arrangements.
- Mixed methods developmental evaluation approaches designed to engage stakeholders and provide action research data on barriers and enablers prior to implementation, and cost effectiveness and feasibility of overall approach.
- Indigenous research excellence criteria inform ethics and dissemination protocols.
