# Supplementary material for: Replanting the Birthing Trees to support Aboriginal and Torres Strait Islander parents and babies: protocol for developmental evaluation of a comprehensive culturally responsive, trauma-aware, healing-informed, continuity of care(r) model
Source: Front Public Health. 2026 Jan 28;13:1721107. doi: 10.3389/fpubh.2025.1721107 (PMC12891200; doi:10.3389/fpubh.2025.1721107)

VIC PROJECT TIMELINE

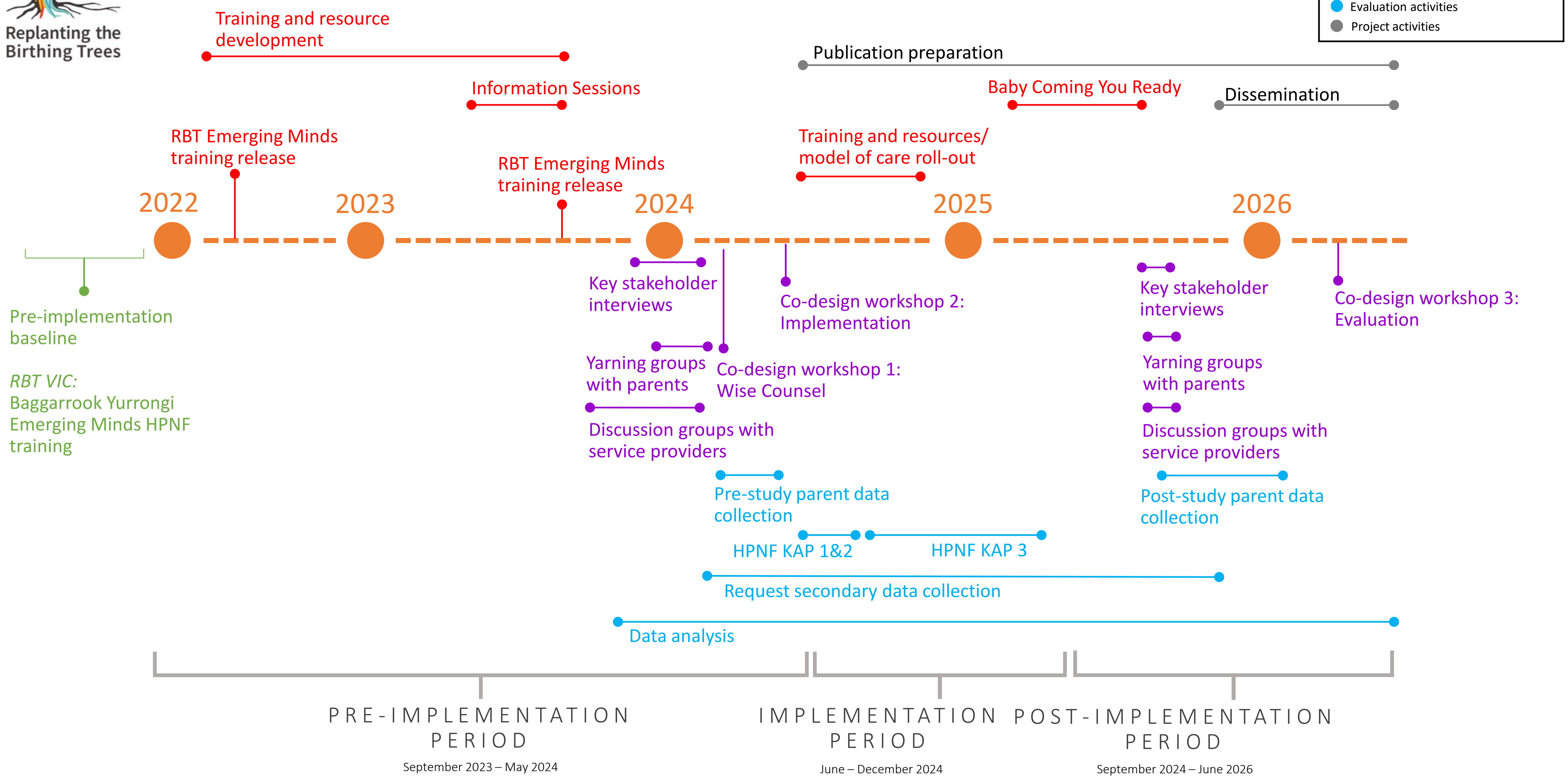

Replanting the Birthing Trees

WA PROJECT TIMELINE

KEY
- Pre-implementation baseline projects
- Implementation activities
- Implementation/Evaluation activities (Action research)
- Evaluation activities
- Project activities

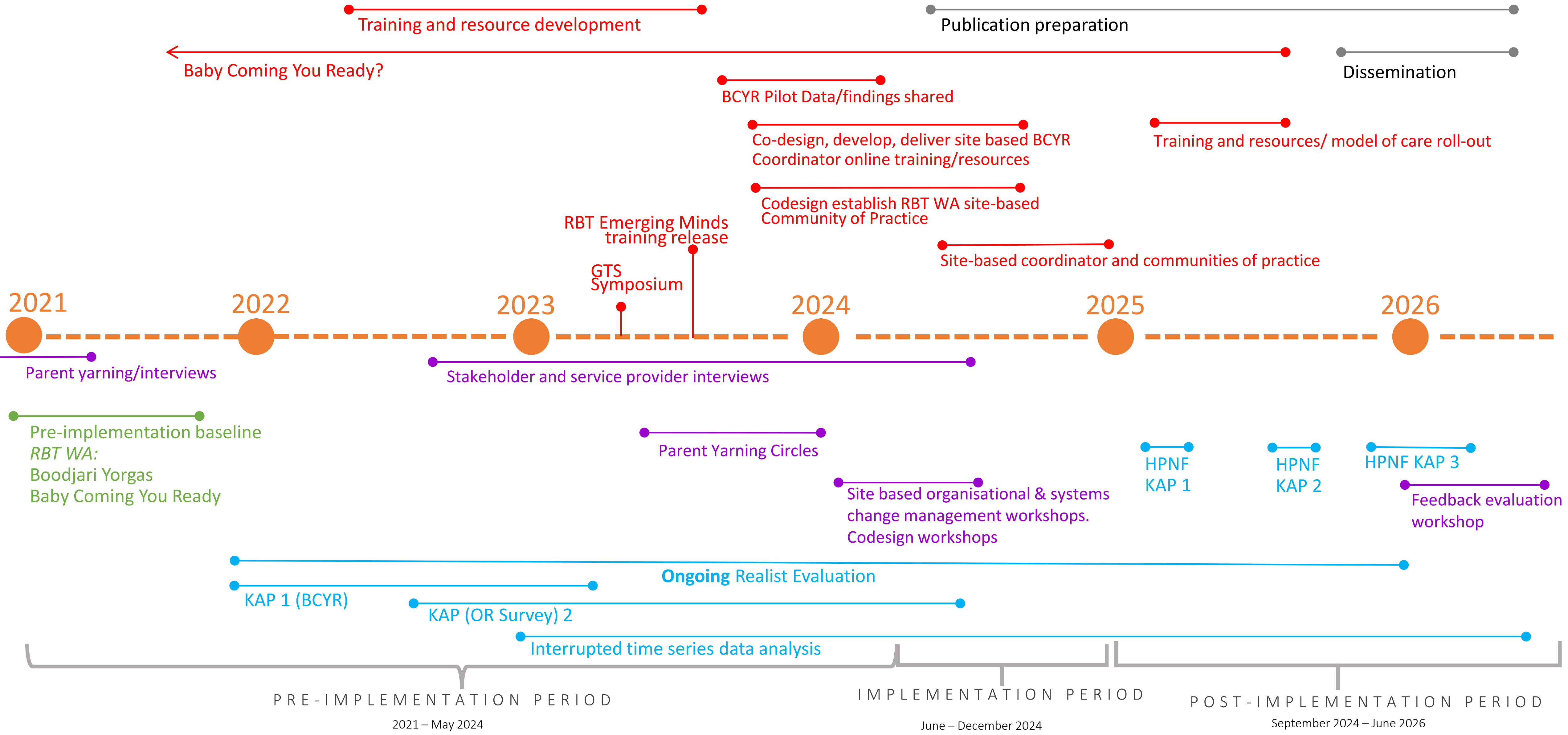

Supplement: Supplementary file 1 [file Data_Sheet_1.pdf]
