# Supplementary figures and images for: Replanting the Birthing Trees to support Aboriginal and Torres Strait Islander parents and babies: protocol for developmental evaluation of a comprehensive culturally responsive, trauma-aware, healing-informed, continuity of care(r) model
Source: Front Public Health. 2026 Jan 28;13:1721107. doi: 10.3389/fpubh.2025.1721107 (PMC12891200; doi:10.3389/fpubh.2025.1721107)

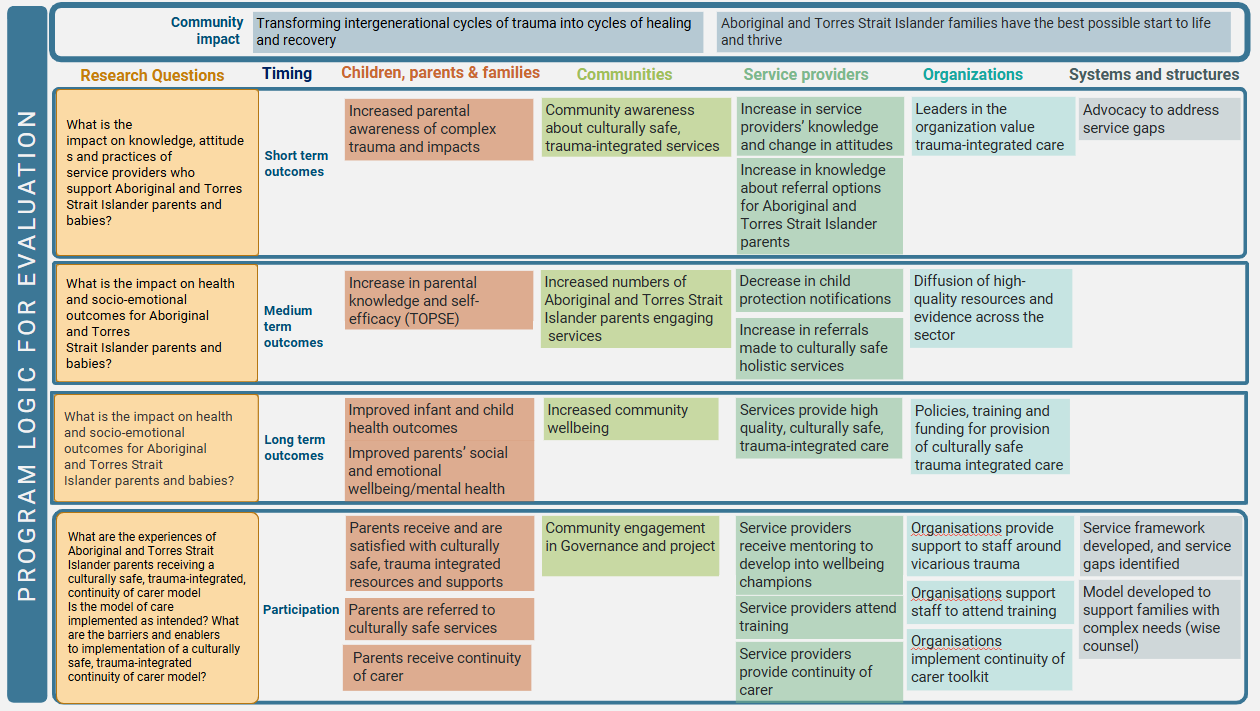

Supplement: Supplementary file 2 [file Table_1.docx]
